# Spatial and temporal metagenomics of river compartments reveals viral community dynamics in an urban impacted stream

**DOI:** 10.3389/frmbi.2023.1199766

**Published:** 2023-08-09

**Authors:** Josué Rodríguez-Ramos, Angela Oliverio, Mikayla A. Borton, Robert Danczak, Birgit M. Mueller, Hanna Schulz, Jared Ellenbogen, Rory M. Flynn, Rebecca A. Daly, LeAundra Schopflin, Michael Shaffer, Amy Goldman, Joerg Lewandowski, James C. Stegen, Kelly C. Wrighton

**Affiliations:** ^1^ Department of Soil and Crop Sciences, Colorado State University, Fort Collins, CO, United States; ^2^ Department of Biology, Syracuse University, Syracuse, NY, United States; ^3^ Biological Sciences Division, Pacific Northwest National Laboratory, Richland, WA, United States; ^4^ Leibniz Institute of Freshwater Ecology and Inland Fisheries, Berlin, Germany; ^5^ Humboldt University, Berlin, Germany

**Keywords:** phage, time-series, auxiliary metabolic genes, hyporheic zone, genome, biogeochemistry, stability, biogeography

## Abstract

Although river ecosystems constitute a small fraction of Earth’s total area, they are critical modulators of microbially and virally orchestrated global biogeochemical cycles. However, most studies either use data that is not spatially resolved or is collected at timepoints that do not reflect the short life cycles of microorganisms. To address this gap, we assessed how viral and microbial communities change over a 48-hour period by sampling surface water and pore water compartments of the wastewater-impacted River Erpe in Germany. We sampled every 3 hours resulting in 32 samples for which we obtained metagenomes along with geochemical and metabolite measurements. From our metagenomes, we identified 6,500 viral and 1,033 microbial metagenome assembled genomes (MAGs) and found distinct community membership and abundance associated with each river compartment (e.g., *Competibacteraceae* in surfacewater and *Sulfurimonadaceae* in pore water). We show that 17% of our viral MAGs clustered to viruses from other ecosystems like wastewater treatment plants and rivers. Our results also indicated that 70% of the viral community was persistent in surface waters, whereas only 13% were persistent in the pore waters taken from the hyporheic zone. Finally, we predicted linkages between 73 viral genomes and 38 microbial genomes. These putatively linked hosts included members of the *Competibacteraceae*, which we suggest are potential contributors to river carbon and nitrogen cycling via denitrification and nitrogen fixation. Together, these findings demonstrate that members of the surface water microbiome from this urban river are stable over multiple diurnal cycles. These temporal insights raise important considerations for ecosystem models attempting to constrain dynamics of river biogeochemical cycles.

## Introduction

Rivers are key modulators of global biogeochemical cycles and provide a dynamic, moving passageway between terrestrial and aquatic ecosystems (Allen and Pavelsky, 2018). Corresponding to ~7% of global CO_2_ and ~5% of global CH_4_ emissions per year, rivers contribute up to 2,508 Tg yr^-1^of carbon dioxide (CO_2_), and ~30.5 Tg yr^-1^ of methane (CH_4_) (Villa et al., 2020; Rosentreter et al., 2021; Friedlingstein et al., 2022; Liu et al., 2022). Microbial communities are key orchestrators of carbon and nitrogen transformations in rivers, where they contribute between 40-90% of hyporheic zone respiration (Pusch and Schwoerbel, 1994; Naegeli and Uehlinger, 1997; Rodríguez-Ramos et al., 2022). Despite a general understanding of the importance of microbial metabolism, river viral communities and their impacts on microbial communities remain poorly described.

Viruses are the most abundant organism on the planet, with estimates of up to 10^31^ viral particles worldwide (Hendrix et al., 1999; Munn, 2006; Bar-On et al., 2018; Mushegian, 2020). These viral predators are mostly studied in marine ecosystems, where viruses can lyse 20-40% of bacteria daily (Weinbauer, 2004; Weinbauer and Rassoulzadegan, 2004; Suttle, 2007; Chow and Suttle, 2015; Guidi et al., 2016) and play key roles reprogramming their bacterial hosts with ecosystem-wide consequences (Sullivan et al., 2006; Anantharaman et al., 2014; Hurwitz and U’Ren, 2016). Although research has mostly focused on marine ecosystems, recent efforts have been made to expand our knowledge of natural viral communities in freshwater aquatic environments like lakes (Roux et al., 2017; Berg et al., 2021) and estuaries (Hewson et al., 2001; Cissoko et al., 2008). Early studies in these systems have shown viral like particle (VLP) abundances and viral productivity (i.e., the number of viruses produced per hour) in rivers can be equivalent, or higher, than those in marine systems (Peduzzi and Luef, 2008; Corinaldesi et al., 2010; Rowe et al., 2012; Peduzzi, 2016). Additionally, early river studies found that up to 80% of bacterial isolate strains from sediments had virulent phage that could be isolated (Lammers, 1992). Together, these foundational works highlight the importance of viral predation in regulating microbial dynamics in river ecosystems.

There are two key reasons why it remains difficult to link viral communities to river ecosystem function. First, river microbiome studies are rarely genome-resolved, both from a bacterial and viral perspective. While there is still much to explore, most information on aquatic virus dynamics pertains to oceanic studies (Vincent and Vardi, 2023), and rivers are described as one of the most underexplored aquatic ecosystem with metagenomics, second only to glacier microbiomes (Chu et al., 2020). Although the taxonomic composition of microbial communities in rivers has been well-described by 16S rRNA gene amplicon surveys (Hou et al., 2017; Nelson et al., 2019), it remains unclear how microbial membership relates to relevant ecosystem processes. Likewise, our ability to link the viral community to their respective microbial hosts, and subsequently to ecosystem biogeochemistry, remains hindered by a lack of genome-resolved studies. Second, river studies are often not temporally constrained. Although significant changes in river chemistry and hydrology are observed at seasonal periods (Tomalski et al., 2021), they are also known to change at sub-daily scales (Lundquist and Cayan, 2002; Alonso et al., 2017), particularly in human-impacted rivers affected by wastewater treatment plant effluent and reservoirs (Luo et al., 2020; Wang et al., 2021; Lu et al., 2022). This is particularly important when considering the microbial component of river systems, as microbial generations are on the scale of minutes to hours, and microbiomes can shift metabolically in hours (Wang et al., 2015; Erbilgin et al., 2017; Gibson et al., 2018). Nonetheless, river microbiome time-series are often resolved at seasonal scales (Kaevska et al., 2016; Malki et al., 2021), meaning our understanding of viral and microbial community dynamics across relevant temporal gradients (i.e. hours) remains poorly understood.

**Figure 1:**
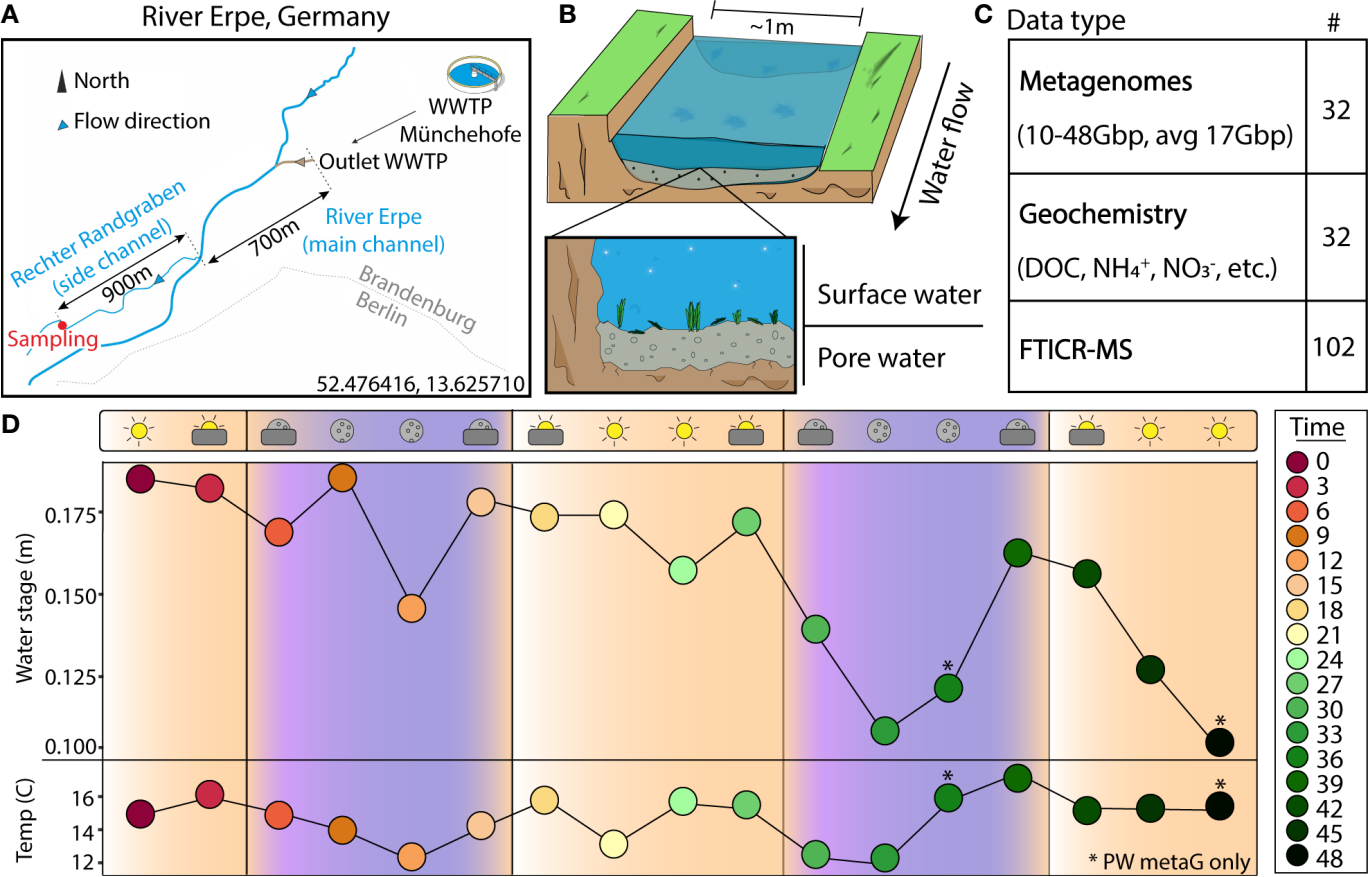
Experimental design enables a genome- and time-resolved view of microbial communities at a finely scaled resolution. **(A)** River Erpe sampling site that is located near Berlin, Germany. **(B)** Conceptual schematic of the surface and pore water compartments that were sampled as part of this research. **(C)** Table of data types that were collected as part of this sampling effort. **(D)** Sampling schematic over 48-hour period with two ecological variables (water stage, and temperature) shown across the timepoints collected. The colors and icons highlight the hour of the day when samples were collected. Asterisks (*) denote samples where only pore water metagenomes were collected

To address these knowledge gaps, we collected a finely resolved metagenomic time-series at the River Erpe near Berlin, Germany, a lowland river receiving treated wastewater. Our sampling campaign included biogeochemical measurements every 3 hours for 48 hours across both surface water (SW) and pore water (PW) compartments that were paired to metagenomics and metabolomics (**Figures 1A–D**). This study design provided a metagenomically resolved dataset which enabled us to interrogate how viral and microbial communities are structured across river compartments, and how this metabolic potential could modulate biogeochemical processes. Additionally, the temporal resolution of our dataset allowed us to analyze both the persistence of viral and microbial communities across compartments, as well as the individual genome stability throughout the 48 hours of sampling. Finally, by using genome-resolved metagenomics, we show that viruses can be linked to hosts in river ecosystems, and that these linkages can reveal putative interactions that may be relevant to understanding the temporal dynamics of ecosystem biogeochemistry.

## Methods

### Sample collection, DNA isolation, and chemical characterization

The River Erpe is highly influenced by diurnally fluctuating effluent volumes of the Münchehofe wastewater treatment plant and consists of up to 80% treated wastewater (Mueller et al., 2021). Our sampling site is in a side channel with a mean discharge of 25 l/s (Lewandowski et al., 2011; Mueller et al., 2021) (**Figure 1A**). For sample collection, a sampling station was set up ~1m from the shoreline of the River Erpe side channel “Rechter Randgraben” (52.476416, 13.625710), 1.6km from the wastewater treatment plant outlet leading the same water as in the main channel as previously described (Mueller et al., 2021), and in accordance to the Worldwide Hydrobiogeochemistry Observation Network for Dynamic River Systems (WHONDRS) protocol (Stegen and Goldman, 2018). Samples were collected on September 25, 2018. More information on the River Erpe sampling methods can be found in another publication from our team, as well as the original public data repository (Wells et al., 2019; Mueller et al., 2021). Briefly, for surface water (SW), 60ml at a time of SW were collected manually with a syringe and tubing fixed in the water column and then passed through a 0.20μm filter until clogged. A cap was then put on the filter, filled with 3ml RNAlater, and refrigerated until extraction. For pore water (PW), 60ml of PW from 25cm sediment depth were collected with a stainless-steel rod in the middle of the channel. The rods were covered with a filter mesh sock over the screened area at the tip, pushed into the sediment, and equipped with a Teflon suction line. Samples were then taken by manually pulling 60ml of PW with syringes attached to the suction line and filtering them through a 0.20μm filter until clogged. The filter was then capped, filled with 3ml RNAlater, and refrigerated until extraction. Each of these processes were repeated every 3 hours over a period of 48hrs in September of 2018, resulting in 15 SW and 17 PW metagenomes. 2 SW samples failed due to lack of biomass. For DNA isolation, filters were cut into ~5mm^2^ pieces and added to the bead bashing tubes of Quick-DNA Soil Microbe Microprep Kit (Zymo). The nucleic acids were then extracted according to the manufacturer protocol and sequenced at the Genomics Shared Resource Anschutz Medical Campus, Colorado. Accession numbers, total metagenomic reads, and sample sizes can be found on **Supplemental Table 1** and the original data repository (Wells et al., 2019).

Chemical characterization was performed as previously described (Mueller et al., 2021). Water samples were filtered with 0.2μm polyethersulfone Sterivex for Fourier transform ion cyclotron resonance mass spectrometer (FTICR-MS) analysis or regenerated cellulose for all other analytes, then acidified to a pH of 2 with 2M HCl and stored at -18°C until analysis. Samples were analyzed at the Leibniz Institute of Freshwater Ecology and Inland Fisheries for nitrate and sulfate (ion chromatography, Metrohm 930 Compact IC Flex), ammonium and soluble reactive phosphorous (SRP) (segmented flow analyzer Skalar SAN, Skalar Analytical B.V., Netherlands), and manganese and iron (inductively coupled plasma optical emission spectrometry (ICP-OES), (ICP iCAP 6000 series, Thermo Fisher Scientific Inc.). Dissolved organic carbon (DOC) concentrations were analyzed via infrared gas analyzer (NDIR) after combustion (TOC/TN Analyzer, Shimadzu). Dissolved organic matter (DOM) data is part of the WHONDRS dataset (Wells et al., 2019) and was analyzed using a 12T Bruker SolariX FTICR-MS (Bruker, SolariX, Billerica, MA, USA) at the Environmental Molecular Sciences Laboratory in Richland, WA. Once peaks were picked using the Bruker data analysis software and formulas were assigned using Formularity (Tolić et al., 2017), DOM was classified into seven compound classes based upon hydrogen to carbon ratio (H:C), and oxygen to carbon (O:C) ratios (Kim et al., 2003). FTICR-MS analysis does not allow for a quantitative approach, therefore compound class data was analyzed qualitatively, and DOM composition was evaluated using the number of molecular formulas in every compound class as described in the original publication (Mueller et al., 2021). The biogeochemical measurements for this study can all be found on **Supplemental Table 1**.

### Metagenome data processing and assembly

Each set of metagenomic reads were trimmed using Sickle v1.33 with default settings (Joshi NA, 2011), and assessed using FastQC (v0.11.2) (Andrews, n.d.). Trimmed reads were then assembled with either 1) metaSPAdes BBCMS pipeline (v3.13.0) (Metagenome Assembly Workflow (v1.0.1) — NMDC Workflows 0.2a documentation, n.d.), 2) Megahit (v1.2.9) (Li et al., 2015), or 3) IDBA UD (v.1.1.0) (Peng et al., 2012). For metaSPAdes pipeline, reads were merged into a single.fa file using fq2fa (Shen et al., 2016). Then, bbcms was run with flags “mincount = 2”, and “highcountfraction = 0.6”, followed by metaSPAdes using kmers 33, 55, 77, 99, 127, and flag “–meta”. For Megahit, reads were assembled with flags “k-min = 31”, “k-max = 121”, “k-step = 10”, and “m = 0.4”. For IDBA_UD, samples were rarefied to 25% of reads using BBMAP’s reformat.sh (Bushnell, 2014) with flags “samplerate = 0.25” and “sampleseed = 1234”. These 25% of subset reads were then merged into a single.fa file using fq2fa (Shen et al., 2016) and then assembled with default parameters. Assembly statistics for each sample can be found in **Supplemental Table 1**.

### Viral identification, taxonomy, and annotations

Viral metagenome assembled genomes (vMAGs) were identified from each set of assemblies using Virsorter2 and CheckV using the established protocols.io methods (Guo et al., 2021a; Guo et al., 2021b). Resulting genomes were then screened based on VirSorter2 and checkV output for viral and host gene counts, VirSorter2 viral scores, and hallmark gene counts (Guo et al., 2021b). Viruses were then annotated with DRAM-v using the “–use_uniref” flag, and further manually curated according to the established protocol (Shaffer et al., 2020; Guo et al., 2021b). The resulting subset of 6,500 viral genomes were clustered at 95% ANI across 85% of shortest contig per MIUViG standards (Roux et al., 2018) resulting in 1,230 viral populations.

Viral taxonomic identification of viral populations was performed using protein clustering methods with vContact2 using default methods (Bin Jang et al., 2019). We supplemented the standard RefSeq v211 database containing 4,533 vMAGs with viral genomes from an additional 303 river and wastewater treatment plant metagenomes that were publicly available from 1) JGI IMG/VR (6,254 vMAGs ≥10kb), 2) two previously unpublished anaerobic digestor metagenomic datasets that were mined in-house (14,436 vMAGs ≥10kb) (https://doi.org/10.5281/zenodo.7709817), 3) a previously published wastewater treatment plant sludge database (7,443 vMAGs ≥10kb) (Shi et al., 2022), 4) a previously available reference database that included freshwater ecosystem viruses (2,032 vMAGs ≥10kb) (Rodríguez-Ramos et al., 2022), and 5) the 43 TARA Oceans Virome datasets (5,476 vMAGs ≥10kb) (Brum et al., 2015). This resulted in an additional 35,641 reference vMAGs in our network. Proteins file for all vMAGs used in the network as well as accession numbers are available on Zenodo (https://doi.org/10.5281/zenodo.7709817). Results from vContact2 can be found in **Supplemental Table 2**.

Viral population genome representatives were annotated using DRAM-v (Shaffer et al., 2020). To identify putative auxiliary metabolic genes (AMGs), auxiliary scores were assigned by DRAM-v to each annotated gene based on the following previously described ranking system: A gene is given an auxiliary score of 1 if there is at least one hallmark gene on both the left and right flanks, indicating the gene is likely viral. An auxiliary score of 2 is assigned when the gene has a viral hallmark gene on one flank and a viral-like gene on the other flank. An auxiliary score of 3 is assigned to genes that have a viral-like gene on both flanks (Shaffer et al., 2020; Rodríguez-Ramos et al., 2022). Genes identified by DRAM-v as being high-confidence possible AMGs (auxiliary scores 1-3) were subjected to protein modeling using Protein Homology/AnalogY Recognition Engine (PHYRE2) (Kelley et al., 2015), and manually verified. All files for vMAG quality and annotations can be found in **Supplemental Table 2**.

### Bacterial and archaeal metagenomic binning, quality control, annotation, and taxonomy

Bacterial and archaeal genomes were binned from each set of assemblies with MetaBAT v2.12.1 (Kang et al., 2019) as previously described (Rodríguez-Ramos et al., 2022). Briefly, reads were mapped to each respective assembly to get coverage information using BBmap (Bushnell, 2014), and then MetaBAT was run with default settings on each assembly after filtering for scaffolds ≥2,500bp. Quality for each MAG was then assessed using CheckM (v1.1.2) (Parks et al., 2015). To ensure that only quality MAGs were utilized for analyses, we discarded all MAGs that were not medium quality (MQ) to high quality (HQ) according to MIMAG standards (Bowers et al., 2017), resulting in 1,033 MAGs. These MAGs were dereplicated using dRep (Olm et al., 2017) at 95% identity, resulting in 125 MAGs. These 125 MQHQ MAGs were annotated using the DRAM pipeline (Shaffer et al., 2020) as previously described (Rodríguez-Ramos et al., 2022). For taxonomic analyses, MAGs were classified using the Genome Taxonomy Database (GTDB) Toolkit v1.5.0 on November 2021 using the r202 database (Chaumeil et al., 2019). Genome quality, annotations, and taxonomy are reported in **Supplemental Table 3**.

### Virus host linkages

To identify virus-host linkages, we used 1) CRASS (Direct Repeat/Spacer based) v1.0.1 (Skennerton et al., 2013), 2) VirHostMatcher (alignment-free oligonucleotide frequency based) v.1.0.0 (Ahlgren et al., 2017), and 3) PHIST (all-versus-all exact matches based) v.1.0.0 (Zielezinski et al., 2021). CRASS protocol and scripts used are described in detail on GitHub (see Data availability). VirHostMatcher was run with default settings, and the best possible hit for each virus was considered only if it had a d2* dissimilarity score of < 0.2. PHIST was run with flag “-k = 25”, and a PHIST hit was considered only if it had a significant adjusted p-value of < 0.05. To be classified as a virus-host linkage, a virus-host pair had to be predicted by the significant consensus of both VirHostMatcher and PHIST or a virus-host pair had to have a CRASS linkage. With this consensus method, CRASS links, which were always considered good hits, agreed across 60% of predictions at the Genus level, 80% of predictions at the Order level, and 87% at the Class level, suggesting high accuracy of consensus-only, non-CRASS linked virus-host pairs. All virus-host predictions are in **Supplemental Table 2**.

### Genome relative abundance and normalization

To estimate the relative abundance of each vMAG and MAG, metagenomic reads for each sample were mapped to a database of vMAGs or MAGs with Bowtie2 (Langmead and Salzberg, 2012) at an identity of 95%, with minimum contig coverage of 75% and minimum depth coverage of 3x. To normalize abundances for known temporal omics data biases (Coenen et al., 2020), we performed a library size normalization of abundance tables using TMM (Robinson and Oshlack, 2010). Given that PW and SW organism abundances were drastically different in magnitude, and that abundance zeroes across compartments are likely real zeroes, vMAGs and MAGs were considered to be present if detectable in at least 10% of samples in either compartment. Organisms detected in > 10% PW samples were labeled “pore”, organisms detected in > 10% SW samples were labeled “surface”, organisms > 10% PW and SW samples were labeled “both”, and organisms that were in < 10% SW and PW samples were removed. Based on these groups, the TMM abundances file was split into two different files, one for PW samples (n = 17) including “pore” and “both” organisms, and one for SW samples (n = 15) including “surface” and “both” organisms. Abundances for vMAGs and MAGs can be found in **Supplemental Table 2**, **3**, and specific commands can be found on GitHub.

### Temporal and statistical analyses

Temporal analyses were all performed in R with the TMM normalized abundances described above. To determine which environmental parameters were significantly driving differences across our compartments, we performed multiple regressions using envfit in the vegan R package (Oksanen et al., 2016) across multiple types of ordinations. Principal Coordinate Analysis (PCA) for biogeochemistry were done with vegan in R. Dissimilarities in community composition were calculated with the Bray-Curtis metric in vegan (Oksanen et al., 2016) for all vMAGs and MAGs that were present in >3 samples per each compartment. Nonmetric multidimensional scaling (NMDS) was then used with k = 2 dimensions for visualization. An analysis of similarity (ANOSIM) was performed using the base R stats package in order to determine community similarity between river compartments. PERMANOVA analyses were done in R using the adonis function from vegan. The NMDS ordinations of the vMAGs and MAGs were compared using the PROCRUSTES function in vegan. To visualize the relative contribution of each biogeochemical variable, we calculated the envfit vector using function ordiArrowMul and plotted them using ggplot. Shannon’s H’ were done using TMM normalized values with vegan in R. Species accumulation curves were done using the vegan function specaccum in R. All R code and files are available on GitHub.

To determine the relative stability of surface and pore water communities, we first calculated the differences in Bray-Curtis dissimilarity for each sample and its prior timepoint and then ran an unpaired t test to compare the mean differences across compartments with the vegan package in R. For assigning the persistence of the different genomes, we used previously established metrics to assess persistent (present in ≥ 75% of samples), intermittent (present > 25% <75% of samples), or ephemeral (present in ≤ 25% of samples) categories (Chow and Fuhrman, 2012). For establishing the abundance stability, we assessed the total number of samples in which each individual persistent genome fluctuated by ± 25% of the median relative abundance value across all samples. Then, using the established cutoffs by Fuhrman and Chow et al. (Chow and Fuhrman, 2012)., we categorized our genomes as stable (shifting in ≤ 25% of samples), intermediately stable (shifting in > 25% < 75% of samples) and unstable (shifting in ≥ 75% of samples). Fishers exact test for count data was used for assessing the significance of difference in stability metrics using fisher.test from R base stats package. The enrichment analyses for AMGs were performed using a hypergeometric test between the total AMGs in our dataset and the individual groups of AMGs present in either compartment. The code used is available on GitHub. All temporal analyses and results are in **Supplemental Table 4**.

To reduce the complexity of our microbial data so we could link viral and microbial communities more concretely to ecosystem biogeochemical cycling, we applied a Weighted Gene Correlation Network Analysis (WGCNA) to identify which groups of organisms co-occurred using TMM normalized values in R with package WGCNA (Langfelder and Horvath, 2008; R Core Team, 2018). A signed hybrid network was performed with a combined dataset of MAGs and vMAGs on a per-compartment basis. For SW, we used a minimum power threshold of 14 and a minimum module size of 20. For PW, we used a minimum power threshold of 8 with and a minimum module size of 20. For both networks, a reassign threshold of 0, and a merge cut height of 0.3 were used.

To link the modules to ecosystem biogeochemistry, we performed sparse partial least square regressions (sPLS) on the groups of organisms in each module. sPLS were done using TMM normalized values of co-occurring communities that resulted from WGCNA above in R with package PLS (Chung et al., 2012). Subnetwork membership was related to the overall genome significance for nitrate as described in the WGCNA tutorials document (see GitHub code) using R and the WGCNA package (Langfelder and Horvath, 2008). Full code for WGCNA and SPLS are available on GitHub along with detailed instructions and input files. Visualizations for the AMG and WGCNA figures were made using RawGraphs (Mauri et al., 2017).

## Results

### Metagenomics uncovers viral novelty and biogeography of River Erpe viruses

We sampled 17 pore water (PW) and 15 surface water (SW) metagenomes collected over a 48-hour period using a Eulerian sampling scheme (i.e., at a fixed location) and collected 565.5Gbp of paired metagenomic sequencing (10.2-47.9Gbp/sample, 17.7Gbp avg.) (**Figure 1** and **Supplemental Table 1**). Assembly of these samples revealed 6,861 viral metagenome assembled genomes (vMAGs), of which 6,500 vMAGs were ≥10kb in length and were subsequently clustered into 1,230 species-level vMAGs (**Supplemental Table 2**). The average vMAG genome fragment was 24,164bp (180,216bp max) in the PW, and 19,553bp (153,177bp max) in the SW (**Supplemental Table 2**). Viral MAG richness was consistently 8 times higher (p < 0.01) in the SW (845.0 ± 124.4) compared to the PW (108.3 ± 49.7) and likely drove differences (p < 0.01) in Shannon’s diversity (H’) recorded for the SW (SW = 6.05 ± 0.17, PW = 3.67 ± 0.49) (**Supplemental Figure 1**). In addition to our vMAGs, we identified 1033 metagenome assembled genomes (MAGs) that were dereplicated at 95% identity into 125 medium and high-quality genome representatives. Similarly, MAG richness was higher (p < 0.01) in the SW (SW = 62.6 ± 7.2, PW = 21.8 ± 9.0), and showed significantly different patterns (p < 0.01) in terms of Shannon’s (H’) (SW = 2.9 ± 0.17, PW = 2.6 ± 0.3) (**Supplemental Figure 1**).

**Figure 2:**
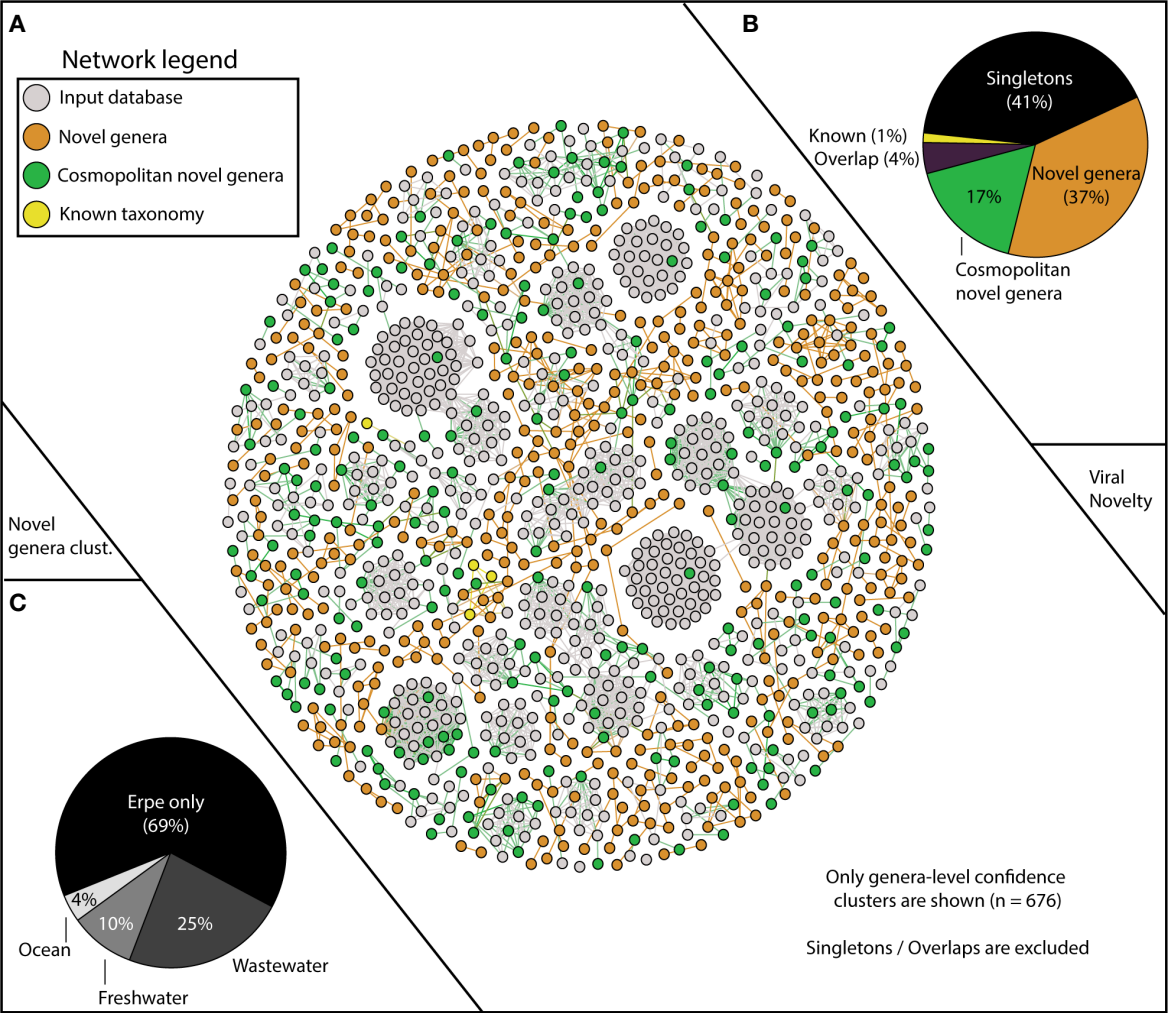
vContact2 reveals Erpe vMAG database constitutes mostly novel genera, and a portion of these are cosmopolitan. **(A)** vContact2 protein cluster (PC) similarity network where nodes represent vMAGs and edges show similarity across edges. Only high-confidence genera-level clusters are shown (n=676) with node color representing whether the vMAG pertains to our input databases (gray) or other categories assigned to vMAGs recovered here: orange shows novel genera (clustering only with Erpe genomes), green shows cosmopolitan novel genera (clustering with viruses from additional input database not from RefSeq), and yellow represents vMAGs with known taxonomy (clustering with known RefSeq vMAGs). Singletons (genomes that do not cluster with any other genomes) are excluded from the visualization (n=518). **(B)** Pie chart shows the distribution of the different categories from the vContact2 network of vMAGs recovered. “Overlap” refers to a category where vContact2 assigns a vMAG to more than one cluster but cannot confidently place in either. **(C)** Pie chart shows the proportion of vMAGs from novel genera in this study that were clustering with vMAGs from different environmental input databases.

Viruses from freshwater systems are not well sampled in the databases commonly used for taxonomic assignment in viral studies (Elbehery and Deng, 2022). To determine the extent of novel viral diversity recovered, we mined additional set of 21,022 vMAGs from a variety of freshwater, wastewater, and marine samples and added this to the original vContact2 database (**Supplemental Table 2**, see **Materials and Methods**). We then performed protein clustering of our unique 1,230 viruses with this modified aquatic database, revealing 3,030 viral clusters (VCs). This network was composed of 19,623 nodes with 679,402 edges, which was simplified to only show protein clusters that contained at least 1 vMAG from this study (**Figures 2A–C**).

Of our 1,230 vMAGs, 1% clustered to known taxonomic representatives of the Caudovirales Order (8 Podoviridae, 7 Siphoviridae, 3 Myoviridae). Of the remaining vMAGs, 37% clustered only to Erpe viruses, constituting 189 novel genera. An additional 41% did not cluster to any vMAG in our database and were “singletons” or “outliers”. Interestingly, 17% of our total vMAGs and nearly half of our novel genera were cosmopolitan in aquatic ecosystems, meaning that while these vMAGs failed to cluster with taxonomically known strains, they did cluster with vMAGs recovered from other ecosystems (**Figure 2B**). Specifically, our cosmopolitan novel genera clustered with vMAGs from wastewater treatment plant sludge or effluent (n=168), other rivers surface or sediment samples (n=65), and marine samples of the TARA oceans dataset (n=25) (**Figure 2C**). Notably, adding these additional viral genomes reduced the total number of River Erpe vMAGs that were categorized as singletons or outliers, resulting in the addition of 49 novel genera.

### Viral and microbial River Erpe microbiomes are compartment-specific

**Figure 3:**
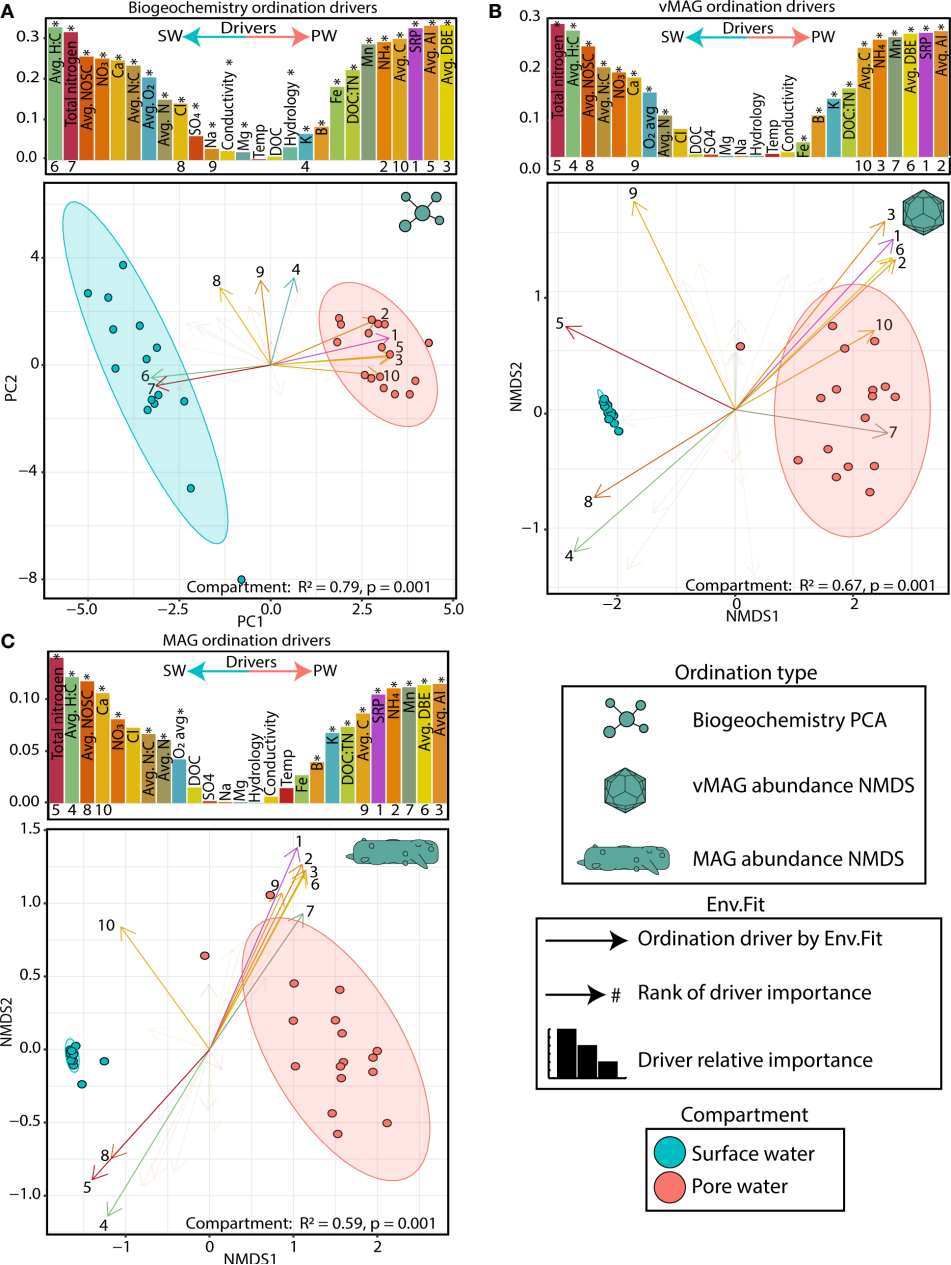
Surface and pore water compartments have distinct viral communities and distributions are driven by biogeochemistry **(A)** PCA plot of biogeochemical measurements where loadings and bars show the biogeochemical drivers per compartment. The size of bars represents the distance between the end of a loading arrow and the center of the plot. Within each bar plot, the drivers are labeled, and asterisks denote significant drivers by env.fit. The top 10 most significant drivers are numbered below each bar and are shown with solid, numbered arrows within the ordination below. **(B)** NMDS ordination of river pore water and surface water vMAG abundances with bars and arrows showing the same as in **(A)**. **(C)** NMDS ordination of river pore water and surface water MAG abundances with bars and arrows showing the same as in **(A)**. Non-compound abbreviations are: nominal oxidative state of carbon (NOSC), calcium (Ca), chlorine (Cl), sodium (Na), magnesium (Mg), dissolved organic carbon (DOC), soluble reactive phosphorous (SRP), aromaticity index (AI), and double bond equivalents (DBE). Note: NOSC values are plotted as the absolute value per value per sample (i.e., a higher SW NOSC driver value translates to a more negative NOSC measurement).

The collected biogeochemistry was significantly structured across compartments and explained a large portion of the total variation in our samples (R^2 =^ 0.79, p < 0.01) (**Figure 3A**). The surface water compartment was driven mostly by 1) the accumulation of alternative terminal electron acceptors (i.e., nitrate (NO_3_^-^), and sulfate (SO_4_^2-^)), 2) the availability of nitrogen compounds (i.e., total nitrogen, avg. N), and 3) a more negative overall nominal oxidative state of carbon (NOSC) and a higher H:C ratio. Conversely, the pore water was characterized by 1) accumulation of NH_4_, 2) the availability of soluble reactive phosphorous (SRP), and 3) the overall concentration of carbon (avg. C), its aromaticity index (AI), and the quantity of double bond equivalents per molecule (DBE). In summary our data indicated more oxidative conditions in the SW (Mueller et al., 2021) while the FTICR-MS data showed that SW carbon was likely more labile, accessible, and thermodynamically favorable.

To determine how viral and microbial communities were structured across these biogeochemical gradients, we recruited the time-series metagenomic reads to our viral database of 1,230 dereplicated vMAGs and 125 MAGs and then performed non-metric multidimensional scaling (NMDS) ordinations (**Figures 3B, C**). Like the geochemical PCA plots, PERMANOVA analyses showed that river compartment explained 67% (p < 0.01) and 59% (p < 0.01) of the variation in viral and microbial communities, respectively. The drivers of both viral and microbial communities were nearly identical in both magnitude and direction. Similarly, a PROCRUSTES analyses showed that vMAG and MAG ordinations are highly coordinated with each other (sum of squares = 0.027, corr. = 0.99, p < 0.01) (**Supplemental Figure 2**) emphasizing the expected dependencies between our identified viral and microbial communities due to our methods. Further highlighting these compartmental distinctions, the abundances of 85% of vMAGs (n = 1051) and 67% of MAGs (n = 87) were indicators of only one compartment (**Supplemental Table 5**). Interestingly, across both viral and microbial ordinations as well as our PCA, time only explained an additional 4-5% of the total variation, albeit significantly (p = 0.03, p = 0.02, and p < 0.01, respectively), likely due to long travel times and hydrological separation (**Supplemental Table 5**).

### Temporally resolved metagenomics unveils compartment-level stability and persistence of viral and microbial communities of the River Erpe

**Figure 4:**
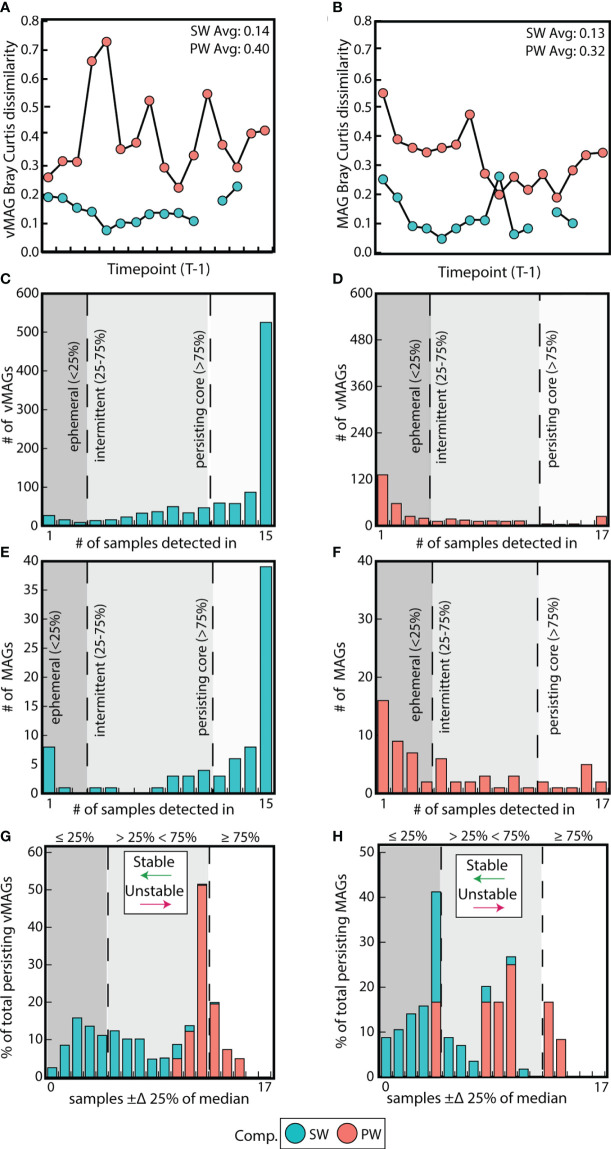
Surface water communities are more stable and persistent than pore water communities. **(A)** Difference in Bray-Curtis dissimilarities between each sample and its prior timepoint calculated for vMAGs and **(B)** MAGs per compartment. **(C)** Bar plots show the number of persistent, intermittent, and ephemeral vMAGs in the SW and **(D)** the PW. **(E)** Bar plots show the number of persistent, intermittent, and ephemeral MAGs in the SW and **(F)** the PW. **(G)** Bar plot where the x-axis shows the number of samples where each vMAG that fluctuates above or below 25% of their median values and the y-axis shows the normalized total percentage of persistent genomes per each compartment that are fluctuating. **(H)** Identical bar plots to those in **(G)** but for MAGs.

SW metagenomic temporal samples for both vMAGs and MAGs were on average 2-fold more similar than PW by Bray-Curtis dissimilarities (BC) (vMAG t = 6.3; MAG t = 6.2, p < 0.01) (**Figures 4A, B**). We next evaluated whether the individual temporal persistence of the viral and microbial genomes shared similar patterns to the BC across compartments, and categorized members using persistence metrics that were previously established (Chow and Fuhrman, 2012). Briefly, if a viral genome was in more than 75% of the samples it was designated as persistent, between 25-75% of samples it was intermittent, and in less than 25% it was ephemeral. Of the 1,035 vMAGs detected in the SW compartment, 70% were categorized as “persistent”, with the remainder being 25% intermittent and 5% ephemeral. Contrastingly, of the 374 vMAGs detected in the PW, only 11% were categorized as persistent, with the remainder being 26% intermittent and 63% ephemeral (**Figures 4C, D**). Similarly, the bacterial and archaeal MAGs shared comparable persistence patterns across the compartments (**Figures 4E, F**). Combined, these results showed that SW communities were less temporally dynamic in terms of BC and had more persistently sampled genomes than the PW.

We then assessed whether the relative abundance of persistent genomes was also temporally stable. Based on a prior study (Chow and Fuhrman, 2012), we tallied the number of samples in which persistent vMAG and MAG relative abundances exceeded ± 25% of their respective median (**Figures 4G, H**, **Supplementary Table 4**). Our results showed that both the relative abundance of vMAGs and MAGs in the SW fluctuate less over time than the PW as shown by Fishers exact t test (p < 0.01). Our persistence and temporal stability results supplement the observation that surface water communities in this urban stream change less over the 48-hour period than pore water communities which are more dynamic.

### Genome-resolved virus-host analyses demonstrated viruses could infect highly abundant, phylogenetically diverse microbial genomes

**Figure 5:**
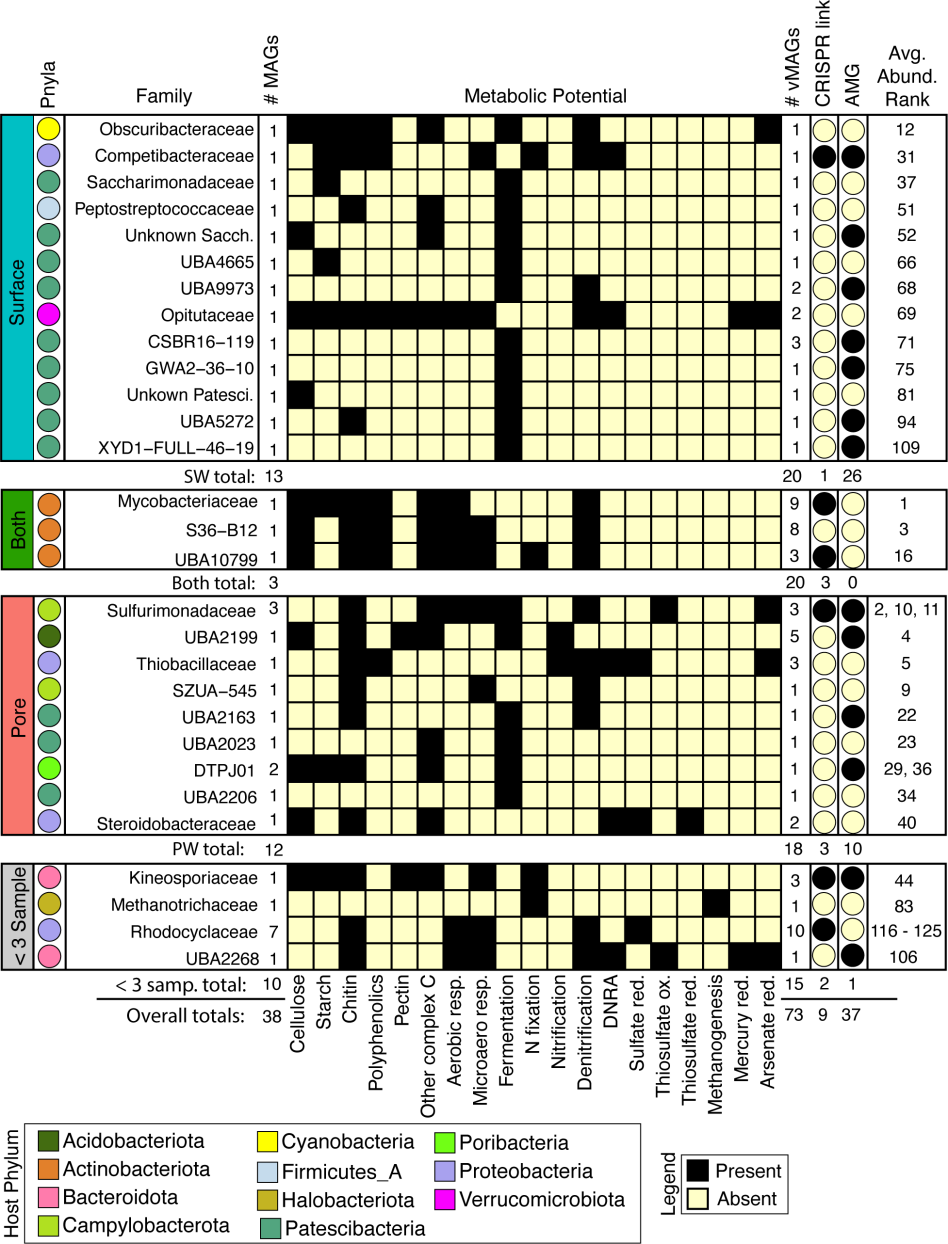
Viruses infect abundant microorganisms in rivers which can influence aerobic and anaerobic C, N, and S cycling by predation or auxiliary metabolic genes. MAG families that had a linkage to a virus are shown and split into their compartment-level distributions. From left to right: Colors of each circle on the leftmost side represent the Phyla, and for each family the total number of MAGs are shown. The presence absence heatmap describes the metabolisms of each family. Following the heatmap are the number of vMAGs that are linked in each family, whether the virus-host link is predicted by CRISPR or consensus method, and if at least 1 infecting vMAG with an AMG is reported. Numbers below each bounding box show totals of above criteria. The overall average rank of each MAG within a family is shown in the rightmost column.

We were able to predict hosts for 73 vMAGs, matching 30% (n = 38) of our total microbial genomes to a viral partner (**Figure 5**). A majority (62%) of vMAGs with host associations were from the SW compartment, with 22% of host-associated vMAGs found in the PW, and around 10% found across both compartments. MAGs that had viruses linked to them were highly abundant, with 54% of our linked vMAGs infecting hosts of the top 25% most abundant MAGs. At the phylum level, 11 of the 20 identified phyla had evidence for a viral host. Notably, all the phyla that could not be assigned a viral link had 2 or less MAG representatives, with the exception of *Desulfobacterota* which had 6 MAGs. Additionally, of the 51 *Patescibacteria* MAGs we recovered in this study, we uncovered 12 possible viral genome links, which to our knowledge is one of the few reports of possible infective agents for members of this phylum (Holmfeldt et al., 2021; Trubl et al., 2021), and is the only one thus far reported in rivers. Ultimately, nearly a third of the genera from our MAG database as defined by GTDB were successfully linked to a vMAG, providing further evidence that viral predation is likely pervasive across these river microbial communities.

To decipher the potential impacts that viral predation could have on biogeochemical cycling across the collected timeseries, we metabolically characterized the 38 viral-linked MAGs from our genome-resolved database and saw a wide array of metabolisms spanning ecosystem chemical gradients (**Figure 5**). Across both compartments, viruses were inferred to impact hosts that could modulate both aerobic and microaerophilic metabolism (carbon respiration), as well as anaerobic metabolisms (nitrate reduction, fumarate reduction, fermentation, and nitrogen fixation). For example, vMAGs were predicted to infect hosts with metabolisms such as methanogenesis (e.g., *Methanothrix*), and sulfur metabolisms (e.g., *Sulfurimonas*), which were encoded more predominantly by MAGs in the PW. vMAGs were also predicted to infect members that encoded denitrification pathways which were prevalent in organisms across both compartments (e.g., *Nanopelagicales*). Interestingly, 20% of the vMAGs that infected hosts were cosmopolitan, with representatives identified in other freshwater and wastewater systems (**Supplementary Table S2**). Together, our genome-resolved database of microbial metabolisms and their putatively infecting viruses gives insight into the underpinnings of River Erpe metabolisms, and show that genome-resolved, river microbiome studies can provide critical perspectives for understanding the impact that viruses can have in river ecosystems.

### Virally encoded auxiliary metabolic genes can potentially alter host metabolic machinery in this urban-impacted river

**Figure 6:**
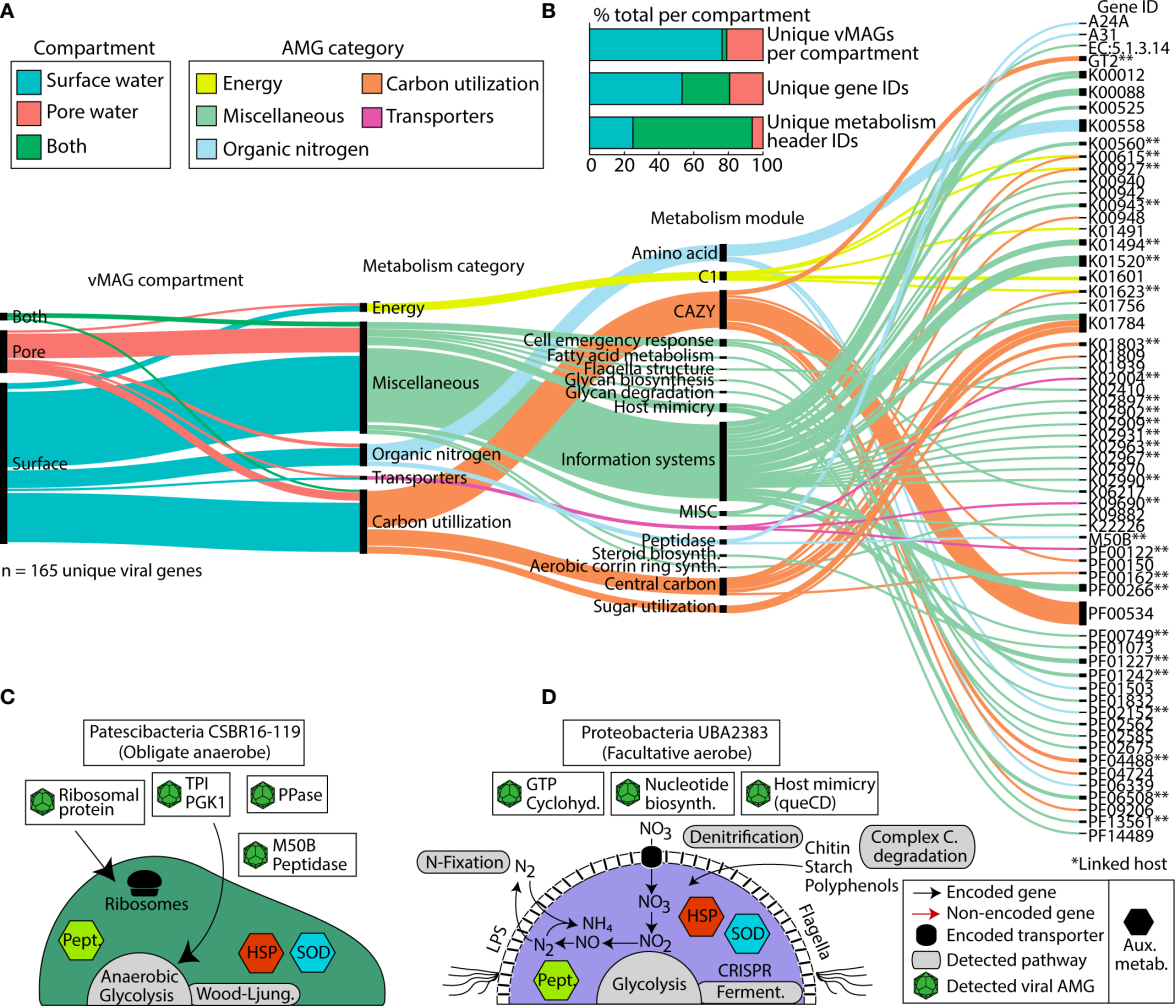
Distribution of viral Auxiliary Metabolic Genes (AMGs) and their function reveals key viral interactions that can enhance host metabolism in river ecosystems. **(A)** Alluvial plot shows the subset of AMGs (77%, n=165) that had a metabolic function annotated by DRAM-v and were 1) not at the end of a contig and 2) did not contain a transposon like element. In the first vertical line, colors show the compartments that each vMAG with an AMG was detected in. The second vertical line shows the different DRAM-v metabolic categories for each AMG. The next vertical line shows the specific metabolic module name as categorized by DRAM. The final line contains each of the Gene IDs for the detected AMGs. Genes that can have multiple functions (n = 13) are duplicated and treated as individual genes within each category. **(B)** Stacked bar charts show the proportion of total AMGs encoded in vMAGs from different compartments at the scaffold, gene ID, and metabolism header ID level as shown in **(A)**. **(C, D)** Genome cartoons of two computationally linked bacterial hosts and their respective metabolisms. Detected viral AMGs are shown as viral icons above each genome cartoon. Pept., peptidases; HSP, heat shock proteins; SOD, superoxide dismutase; queCD, 7-cyano-7-deazaguanine synthase; 6-carboxy-5,6,7,8-tetrahydropterin synthase. Asterisks (**) denote AMGs that were encoded within a virus that had a computationally linked host.

In addition to the impact on microbial communities via predation, viruses can also mediate biogeochemical cycles through enhancing host metabolism with Auxiliary Metabolic Genes (AMGs). We mined our 1,230 vMAGs for putative AMGs and found 165 unique viral AMG candidates after quality filtering, which encompassed 65 unique gene IDs. We failed to see a statistical enrichment for the number of AMGs in either compartment (Fisher’s exact p = 0.77), suggesting their shared importance for the River Erpe. The functionalities of these AMGs at the gene annotation level (e.g., KO number) were mostly conserved across compartments, with only 27% of unique gene IDs present in both compartments. However, at the DRAM-v functional module level (e.g., amino acid metabolism) 69% of metabolisms were present across both our ecological gradients (**Figure 6B**). Conserved DRAM categories across compartments pertained to carbon utilization (e.g., CAZyme inferred substrates (cellulases), glycolysis), energy generation (e.g., CO_2_ fixation (reductive pentose phosphate pathway)), and other reactions (methionine degradation). We note that genes necessary for viral replication like nucleotide biosynthesis, ribosomal proteins, host mimicry, glycan biosynthesis, cofactor and vitamin metabolism, and molecular transporters were conserved between compartments.

There were also some unique AMGs that did show compartment specificity. For example, within the surface water we exclusively detected AMGs for organic nitrogen mineralization and transcriptional regulation (i.e., peptidase M50), sugar metabolism (i.e., fructose and mannose (mannose-6-phosphate isomerase)), and motility (i.e., flagellar motor switch protein *FliG*). We note that among our putative AMGs, we also identified several glycosyltransferases (i.e., GT1, GT2, GT17, and a general sugar binding GT) (**Figure 6A**). These GT genes are commonly reported as carbohydrate degradation enzymes in other studies, particularly those annotated as glycosyltransferase 2 (GT2) because of the breadth of reactions in their CAZyme families. As such, while we report these in our figure and supplemental information for transparency, we urge caution when inferring these activities in carbon degradation.

We next considered AMGs that either expanded the host metabolism or that were complementary to the host metabolism (i.e., Class I AMGs) (Hurwitz and U’Ren, 2016). Of the 12 *Patescibacteria* MAGs that had possible viral genome links, MAG representative CSBR16-119 had two possible vMAG linkages. A comparison of the metabolic capabilities of the host and viral genomes indicated multiple shared genes (**Figure 6C**, **Supplemental Table 2**). For example, a peptidase-like protein (M50) that is inferred transcriptional regulator (Rawlings et al., 2018) was present in both the *Patescibacteria* MAG and its infecting vMAG. Across the length of the open reading frame, these bacterial and viral genes shared 77% and 99% nucleotide and amino acid similarity, respectively (**Supplemental Table 2**). The microbial host genome also had a single copy of ribosome L28 encoded, and two viral genomes putatively infecting this host contained a relevant homolog to L28 (>93% identity, over 90% query coverage) (**Supplemental Table 2**).

A second putatively infected genome was *Proteobacteria* UBA2383 (a novel unclassified *Competibacteraceae*) which had broad metabolic capabilities and was persistent in our samples (**Figure 6D**). This MAG was inferred to be a facultative aerobe encoding genes for aerobic respiration and for denitrification. UBA2383 encoded genes supporting a heterotrophic lifestyle including CAZymes necessary for the degradation of complex carbon substrates (e.g., chitin, starch, and polyphenol) and the enzymatic capacity to utilize these substrates for energy (e.g., glycolysis, tricarboxylic acid cycle). This MAG also encoded the ability to fix nitrogen and denitrify. The two vMAGs that were associated with this genome encoded genes to support host metabolism (e.g., GTP cyclohydrolase) which generates important co-factors for bacterial metabolic processes (**Supplemental Table 2**) (He and Rosazza, 2003). Additional AMGs encoded by infecting viruses could potentially enhance nucleotide biosynthesis (dCTP deaminase, dUTP pyrophosphatase, thymidylate synthase) as well as other viral functions like host mimicry genes (i.e., 7-cyano-7-deazaguanine synthase, 6-carboxy-5,6,7,8-tetrahydropterin synthase) to avoid the CRISPR defense mechanisms encoded within the host *Proteobacteria*. More research using non-homology based methods, as well as expression patterns of these AMGs would help confirm their functionality and activity in this urban-impacted stream.

### Co-occurrence networks elucidate ecological patterns that inform ecosystem biogeochemistry

To link viral and microbial communities more concretely to ecosystem biogeochemical cycling, we leveraged our collected temporal samples and applied a Weighted Gene Correlation Network Analysis (WGCNA) to identify organismal groups that co-occurred over the 48-hour sampling time. Highlighting the clear distinctions in SW and PW compartments, WGCNA analyses could not be reasonably performed simultaneously on a combined dataset (scale free topology model fit max = 0.32 at power = 20). As such, using only microbial and viral genomic abundances from either SW or PW separately, we identified 15 and 4 co-occurring modules in the SW and PW, respectively (**Supplemental Figure 4**). The largest module in both networks (turquoise module) contained 254 genomes in the SW and 71 in the PW. In the SW compartment, the overall modules had an average richness of 66 vMAGs and 5 MAGs, while in the PW they had an average richness of 46 vMAGs and 10 MAGs.

**Figure 7:**
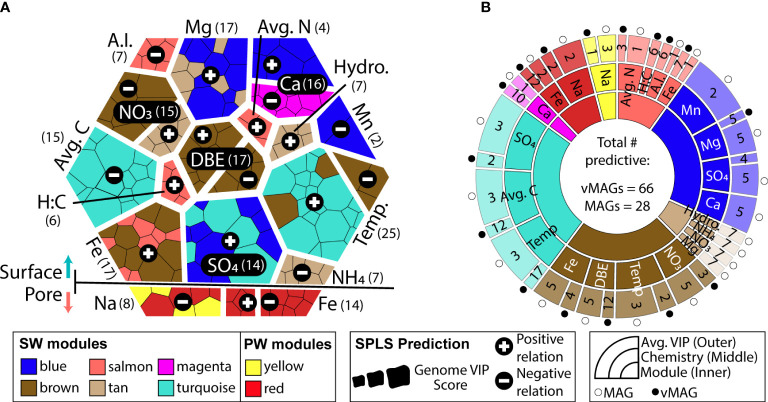
WGCNA co-occurrence networks reveal ecologically similar groups that are related to overall ecosystem biogeochemistry. **(A)** Voronoi diagram shows VIP values of predictions for each predictive genome using a hierarchy structure. Each amorphous square within a group represents a single MAG or vMAG. At the first level (i.e., splitting of the large hexagon into upper and lower groups), SW (top) and PW (bottom) predictions are shown. At the second level (i.e., grouping of individual chemical variables predicted across each compartment), individual chemical variables are shown, per each compartment, and how many vMAGs/MAGs were predictive are denoted by numbers next to each variable name. At the third level (i.e., individual amorphous square or genomes), shapes are sized by the VIP score (>1) of genomes that predict that variable and are colored by their respective WGCNA module. **(B)** Sunburst diagram shows the predictive WGCNA modules in the innermost level, followed by what chemical values each module predicts in the middle level. The outer level shows the average variable importance in projection (VIP) score for each genome type: vMAG (black circles) and MAGs (white circles) for that chemical prediction.

Both surface and pore water communities had modules of co-occurring genomes that were significantly related by sparse partial least square regressions (sPLS) to the collected biogeochemical measurements (R^2^ > 0.3, p < 0.05) (**Figure 7A**, **Supplemental Figure 4**). Only total Fe concentrations were related to modules in both the SW (brown, salmon modules) and PW (red module). SW modules were uniquely related to variables pertinent to nitrogen (nitrate, average total nitrogen), carbon (average total carbon, aromaticity index, hydrogen:carbon), as well as physical (temperature, water stage) and geochemical (magnesium, calcium, manganese, ammonium, sulfate) features in these samples. Of the 8 modules that were significantly related to ecosystem hydrobiogeochemical features, viruses had significant variable importance in projection scores (VIP > 1) in 7 of them, and 70% of the most significantly related genomes across all regressions were viral (**Figure 7B**).

**Figure 8:**
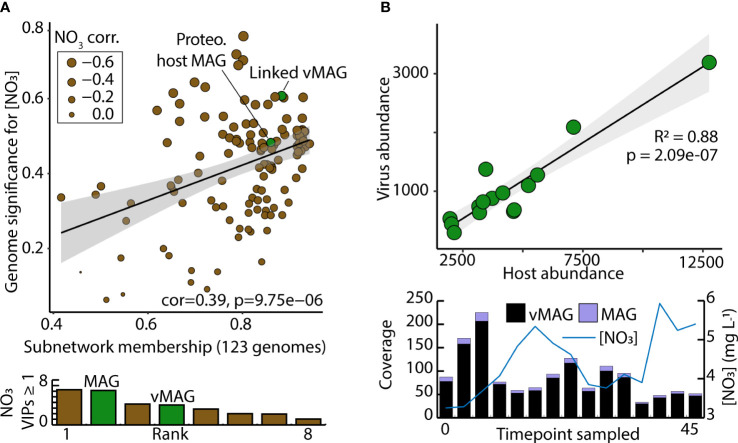
Computationally linked vMAG and MAG pair that share co-occurrence patterns demonstrate high significance for nitrate, and display kill-the-winner dynamics. **(A)** Scatterplot depicts the genomic significance for nitrate of each of the genomes in the brown module in relation to the membership of those genomes within the WGCNA network modules. Below, bar charts show the VIP score (≥1) of the different organisms in the brown module. **(B)** A Virus bacteria ratio (VBR) plot of a viral genome within the brown module that was predicted to infect a Proteobacteria genome. Below it, bar plots show the total coverage across all samples for both the vMAG and the MAG, and a line graph shows the measured nitrate concentrations that these genomes predict.

Of the 73 vMAGs and 38 MAGs that were computationally linked (**Figures 5**, **6**), nearly a quarter of those vMAGs and a third of MAGs were grouped into the same co-occurring modules. Interestingly, the SW brown module was related to the total nitrate concentrations in our dataset and contained a co-occurring virus-host link (**Figure 8A**). The host genome was the *Competibacteraceae* genome in **Figure 6D** and its putatively infecting a virus, which together could play roles in modulating the nitrogen cycling through both fixation and denitrification. This virus and microbial host pair had significant negative correlations to nitrate concentrations and were the second and fourth most significantly related genomes to nitrate within the brown module. The virus bacterial ratio (VBR) for these two organisms was nearly 1:1 and significantly correlated, which is expected of kill the winner dynamics (Trubl et al., 2021), and ultimately highlighting the possible dependency of an infecting vMAG and its host (**Figure 8B**). In support of this relationship, the viral genome coverages were on average 10x more than the putative host MAG coverage, suggesting a possible lytic infection lifestyle. Further underlining the importance of these related genomes, both were designated as persistent (i.e., present in >75% of all collected timepoints) and were the 1^st^ (vMAG) and 9^th^ (MAG) most abundant genomes detected in the surface waters.

## Discussion

### Viral reference databases underrepresent certain habitats, missing cosmopolitan, ecologically relevant lineages

Nearly a quarter of our Erpe viruses formed genus-level clusters with viruses from wastewater and freshwater systems, and of those, 11% encoded a putative AMG with functions for metabolisms such as carbon utilization, organic nitrogen transformations, and housekeeping functions (i.e., transporters and flagellar assembly). While the protein clustering of River Erpe vMAGs to wastewater viruses was not entirely surprising given the sampling location was downstream from the wastewater outlet (Mueller et al., 2021), we note that we also clustered a similar proportion of viruses to other viral genomes from river systems. Notably, this similar clustering proportion for River Erpe viruses was not observed with the TARA ocean viruses (**Figure 2C**, **Supplemental Figure 3**). These results hint at possible ecosystem filtering that may affect the biogeographical patterns of freshwater viruses. Our results also underscore the importance of customized, ecosystem relevant databases in environmental viromics for extending the ecological relevance of these ecosystem modulators, and further understanding the major drivers for river microbiomes.

### Temporally and spatially resolved metagenomics coupled to metabolites and geochemistry enhances our understanding of river microbiome structure

Sampling with a Eulerian method allowed us to detect microbiomes passing through the same space over time in the SW and PW samples. Due to the flow rate of SW, and the potential that PW communities may be more biofilm impacted, we might have expected to see greater microbial and viral changes in the surface compartment than the sediments over the sampled time period. On the contrary, both vMAGs and MAGs were more persistent and had more stable abundance patterns over time in the SW of the River Erpe (**Figures 4A, B**). A possible explanation is that the strong influence of the wastewater treatment plant, where inputs were relatively uniform and continuous over time (Mueller et al., 2021), could contribute to the increased temporal stability we observed. It is also possible that the mixing in the PW hyporheic zone was more frequent than the flow rate within this channel. In support of the former, we did observe strong clustering between our viral genomes and wastewater treatment viral genomes throughout the timeseries (**Figure 2**). Our study is consistent with previous research showing surface water microbiomes are not unstable, or intractable (Graham et al., 2017), and could thus be important for the poorly resolved indices of river health and biogeochemistry that currently exist.

Previous reports using non genome-resolved strategies highlight that richness in river PW and sediments are generally higher than those in the SW for bacterial communities (Abia et al., 2018). Contrary to this, our data shows the opposite trends in the Erpe river for both viral and microbial communities (**Supplementary Figure 1**). One possible explanation could be methodological due to the PW being sampled or assembled less completely as a result of genomic extraction bias caused by fine grain sediments, less sampling volume, or strain level complexity. However, our species area curves did not signify an obvious difference in sampling exhaustion between these compartments (**Supplemental Figure 1**), leaving open the possibility that this finding may be biological.

A possible biological explanation could be that the effluent of the Münchehofe WWTP is altering viral and microbial community diversity. Our geochemical data showed elevated total nitrogen and soluble reactive phosphorous concentrations, which are commonly reported for WWTP impacted systems (Fox et al., 1989; Effler et al., 2010). However, the role of these WWTP influences on river microbiome diversity are variable, with some studies reporting that WWTPs reduce bacterial diversity and overall nutrient concentrations (Atashgahi et al., 2015; Carles et al., 2022; Xie et al., 2022), and other studies showing increases in diversity resulting from eutrophication (Garnier et al., 1992; Marti and Balcázar, 2014).

Our presented and previously published geochemical data inferred more anoxic conditions in the porewater compared to the surface water (Mueller et al., 2021), while the FTICR-MS data indicated a higher concentration of non-labile, microbially inaccessible carbon in the pore water. Additionally, sediment profiles for our samples ranged from 85.8%-96.6% clay content (Mueller et al., 2021), which may impact groundwater and surface water exchanges, resulting in altered nutrient fluxes to inhibit microbial growth (Newcomer et al., 2016; Huettel et al., 1998). Taken together, it is possible that limited nutrient and carbon accessibility contributes to the decreased microbial and viral diversity observed in the PW compared to the SW.

### Viruses have the potential to regulate river biogeochemical cycles by predation and metabolic reprograming of microbial hosts

Although river viral ecology is only recently becoming appreciated, early works suggested that viruses likely play key roles in the structuring of river microbial communities (Peduzzi and Luef, 2009; Peduzzi, 2016). By using a combination of computational methods, we show viruses infect microorganisms that encode a wide array of metabolic functionality critical to river biogeochemistry (e.g., methanogens, denitrifiers, oxygen respirers). In addition to predation, viral auxiliary metabolic genes are recognized across aquatic systems to play key roles in host metabolic reprogramming and can encompass a wide range of processes from photosynthesis to the oxidation of sulfur (Sullivan et al., 2006; Anantharaman et al., 2014). We add to the existing literature and show AMGs in urban river systems may also impact reactions involving nitrogen, carbon, and sulfur cycling. Additionally, one of the vMAGs that was predicted to infect a *Patescibacteria* genome encoded a ribosomal protein, a finding that has been previously reported in other systems for different bacteria (Mizuno et al., 2019). Candidate phyla radiation (CPR) organisms like *Patescibacteria* are present in wastewater treatment plants (Wang et al., 2023) and contain non-redundant, small genomes (Tian et al., 2020; Wang et al., 2023). As such, our results of ribosomal AMGs in *Patescibacteria-*infecting viruses hint at the possibility that viruses may help maintain those small genome sizes by encoding necessary host genes, a concept previously demonstrated for the virus-host dependency of cyanobacterial photosynthesis in oceans (Sullivan et al., 2006).

Other works looking at vMAGs from freshwater lakes and estuaries have shown that some viruses exhibit endemism for certain environments, meaning their distribution is limited to a small geographic area (Ruiz-Perez et al., 2019). This points to an interesting idea that perhaps AMGs may also be tuned to the specific ecological functions of the sampled habitat, and as such that we could expect some degree of endemism in the AMGs. A recent study from an estuary identified significant partitioning of AMG functions between habitat types (water particle and sediment) (Luo et al., 2022). In support of these findings, we identified a subset of unique AMGs within the SW (e.g., flagellar assembly proteins, sugar metabolism) that could potentially be more associated with a lifestyle supported by favorable carbon, and aquatic environments that favor mobility. On the other hand, in the PW we detected AMGs that encoded for plant hemicellulose degradation (**Supplementary Table S2**), an adaptation that could sustain metabolism in a litter impacted, sediment habitat. However, most AMG functional categories from our dataset were highly similar across compartments suggesting some conservation within River Erpe compartments. As such, it is possible that due to the constant mixing of surface and HZ water in this river, and possibly others, stratification at the genomic potential may be less notable, and expression information may be necessary to capture habitat specific differences. Ultimately, this study highlights how moving forward annotation resolution and expanding reference database(s) are important factors to consider when extrapolating AMG inferences across datasets (Hurwitz and U’Ren, 2016; Shaffer et al., 2020).

In conclusion, our results highlight the power of temporally resolved metagenomics in understanding river microbiome dynamics. Leveraging the community-sequenced dataset of the River Erpe, we provide insights regarding compartment-level microbiome stability and show surface water microbiomes may not be as “untraceable” or “unstable” as previously thought. This stability at a genome-resolved view, suggests microbial content could add to the growing body of indicators for river wellness. Ultimately, this research provides a strong scaffolding foundation for future temporally resolved river studies that couple microbial omics measurements to biogeochemical rates to bridge the gap in understand overall ecosystem functionality.

## Data availability statement

The datasets supporting the conclusions of this article are publicly available and collected as part of the Worldwide Hydrobiogeochemistry Observation Network for Dynamic River Systems (WHONDRS) collective sequencing project and are all publicly available on ESS-Dive (Wells et al., 2019). The 125 MAGs are deposited in NCBI BioProject, accession number PRJNA946291, and the 1230 vMAGs have been deposited in NCBI BioProject, under ID SAMN34000891. The 125 MAGs, the 1230 vMAGs, the raw annotations for each genome, and the dataset of freshwater and wastewater viruses we used to cluster to the HUM-V viruses are hosted on Zenodo (https://doi.org/10.5281/zenodo.7709817). All scripts, commands, and input data used for this manuscript are available at https://github.com/jrr-microbio/erpe_river.

## Author contributions

JR-R: Conceptualization, Methodology, Validation, Formal analysis, Data curation, Writing - Original Draft, Writing - Review and Editing, Visualization. AO: Conceptualization, Methodology, Writing - Original Draft, Formal analysis, Writing - Review and Editing, MB: Methodology, Software, Writing - Review and Editing. RD: Methodology, Investigation, Writing - Review and Editing. BM: Investigation, Writing - Review and Editing. HS: Investigation, Writing - Review and Editing. JE: Formal analysis, Writing - Review and Editing. RF: Software, Writing - Review and Editing. RAD: Formal analysis, Writing - Review and Editing. LS: Writing - Review and Editing, MS: Software. AG: Conceptualization, Methodology, Supervision, Writing - Review and Editing. JL: Conceptualization, Methodology, Supervision, Writing - Review and Editing. JS: Conceptualization, Methodology, Supervision, Funding acquisition, Writing - Review and Editing. KW: Conceptualization, Methodology, Resources, Writing - Original Draft, Writing - Review and Editing, Supervision, Project administration, Funding acquisition. All authors contributed to the article and approved the submitted version.
