# Supplementary figures and images for: Spatial and temporal metagenomics of river compartments reveals viral community dynamics in an urban impacted stream
Source: Front Microbiomes. 2023 Aug 9;2:1199766. doi: 10.3389/frmbi.2023.1199766 (PMC12993620; doi:10.3389/frmbi.2023.1199766)

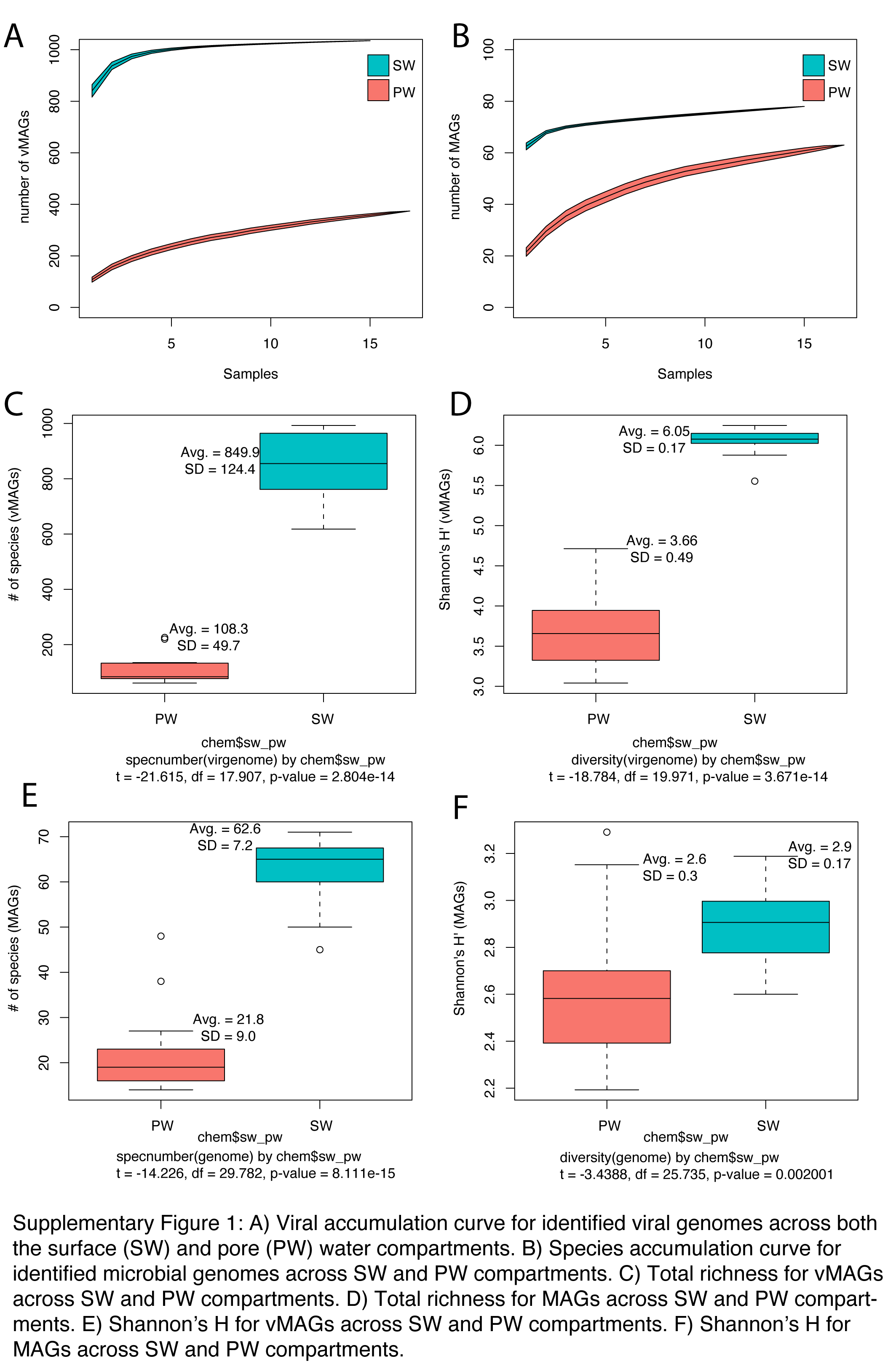

Supplement: Supplementary file 1 [file Image_1.tif]

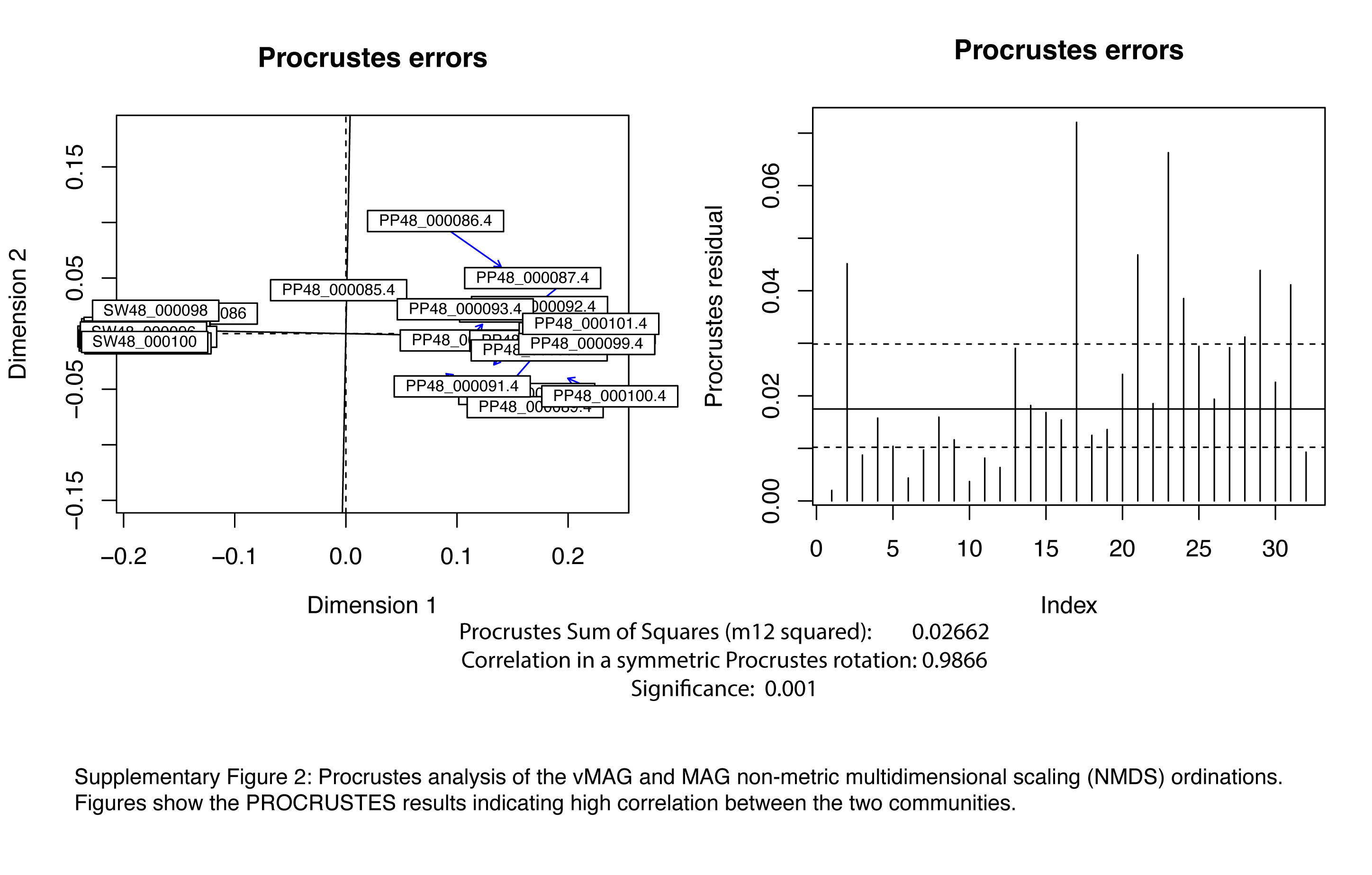

Supplement: Supplementary file 2 [file Image_2.tif]

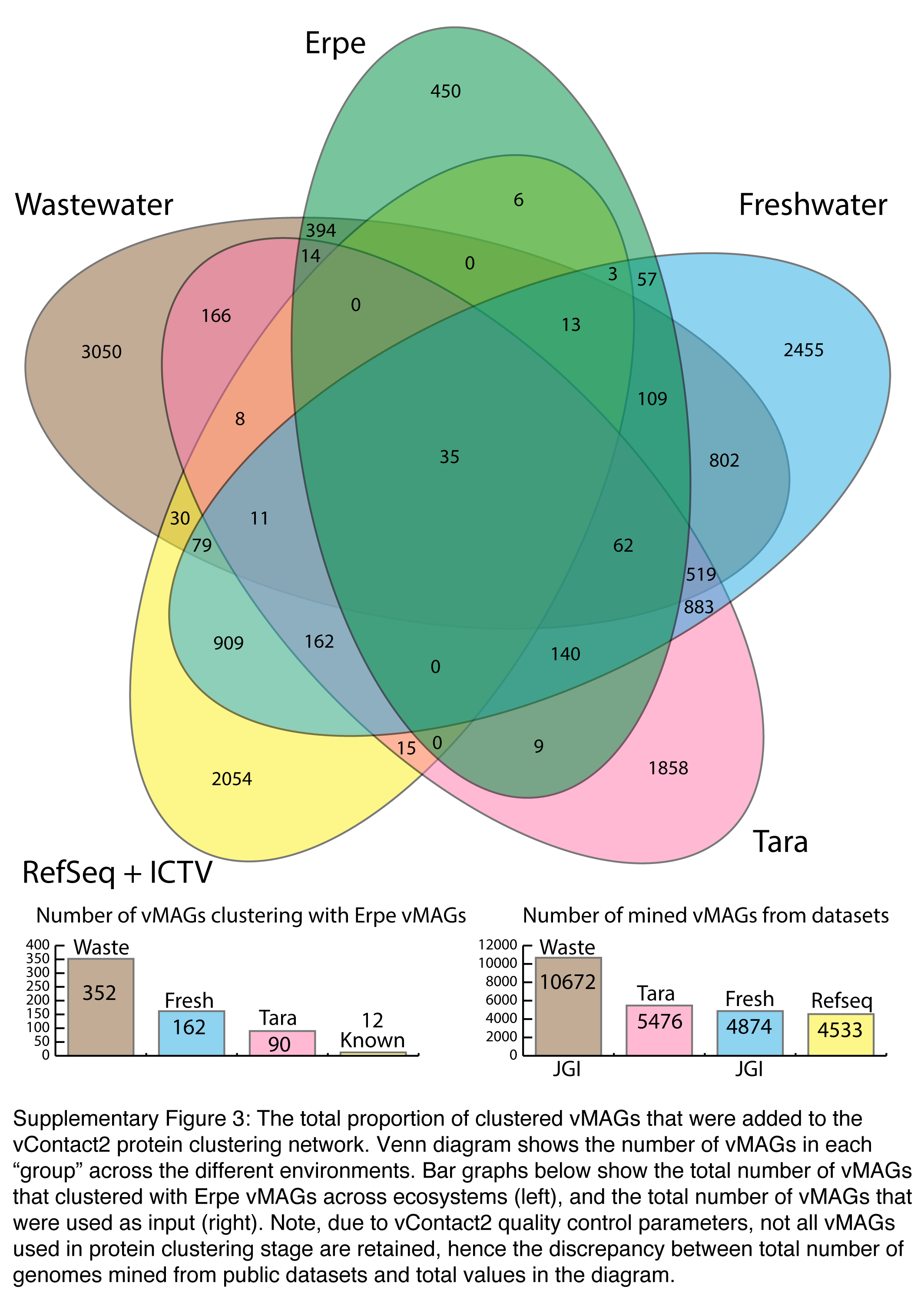

Supplement: Supplementary file 3 [file Image_3.tif]

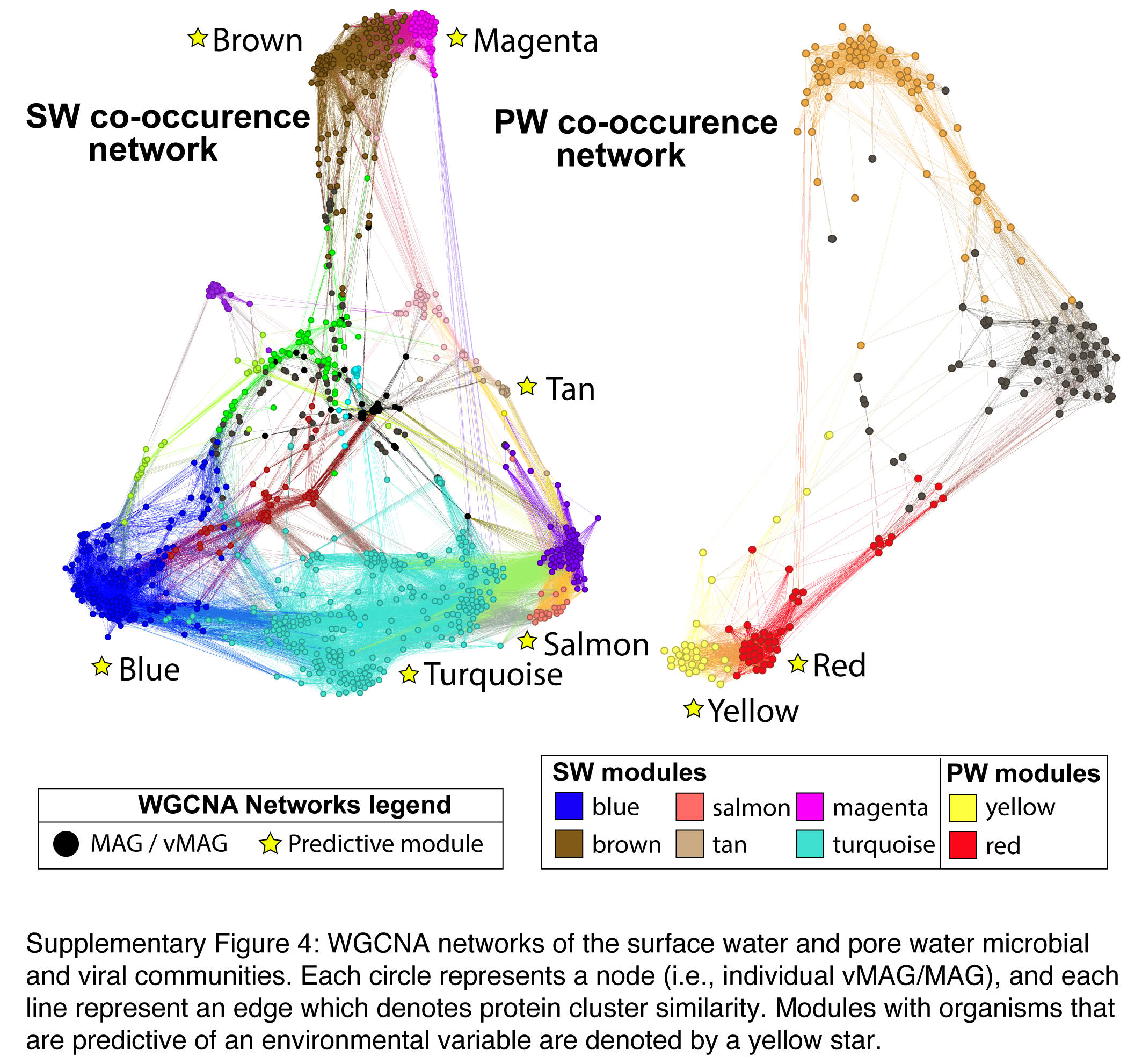

Supplement: Supplementary file 4 [file Image_4.tif]
